# Epidemiological profile and clinical characteristics of metabolic syndrome in Marrakesh, Morocco

**DOI:** 10.11604/pamj.2020.36.133.19913

**Published:** 2020-06-26

**Authors:** Zineb Hannoun, Khouloud Harraqui, Rachmat Attoumane Ben Ali, Kamar Tahiri, Omar Ben Smail, Ilyas Samara, Fatine El Arabi, Abdellatif Bour

**Affiliations:** 1 Laboratoire des Essais Biologiques, Equipe de Transition Alimentaire et Nutritionnelle (ETAN), Faculté de Sciences, Ibn Tofail University, PoBOX: 133, Kenitra 14000, Morocco

**Keywords:** Epidemiology, metabolic syndrome, cardiovascular diseases, central obesity, Morocco

## Abstract

**Introduction:**

the purpose of this study was to bring out some epidemiological and clinical characteristics of metabolic syndrome.

**Methods:**

a total of 300 subjects willingly participated in the present study which was conducted at Ibn Zohr regional hospital in Marrakesh. We were interested in socio-demographic variables, body mass index (BMI) which assesses the degree of obesity of each subject. The blood parameters were determined by an adequate biochemistry automaton. All statistical analyses were performed using SPSS software.

**Results:**

among the 300 subjects who participated in the study, 57.3% were females and 42.7% were males with a sex-ratio of 0.74. The average age was 51.6 ± 13.42 years old. Seventy nine of the participants (26.3%) had a metabolic syndrome, with a predominance of women: 60 women (34.9%) and 19 men (14.8%). Illiterates (33.8%) and married subjects (25.6%) were the most affected by the metabolic syndrome. The high waist circumference found in 97.5% was the predominant criteria in our study. Finally, the statistical analysis showed a significant association between high waist circumference, BMI and the presence of metabolic syndrome (P = 0.001>) and (P < 0.001) respectively.

**Conclusion:**

the metabolic syndrome is slowly but surely setting. Implementing prevention strategies and encouraging healthy lifestyles will surely minimize serious public health problems in the city.

## Introduction

The metabolic syndrome is defined as the association of several metabolic abnormalities in the same individual, namely, abdominal obesity, hypertriglyceridemia, a low HDL cholesterol, hypertension and insulin resistance, causing eventually to three times the cardiovascular risks and to nine times the risk of type 2 diabetes [1]. Fast food is becoming more common in Morocco, favoriting the consumption of high caloric food. This evolution reflects the nutritional transition seen in rural as well as urban areas [2]. Metabolic syndrome is gaining more importance especially with many studies showing the increasing risks of cardiovascular diseases. However, the studies devoted to this subject in Morocco, are lacking, especially the studies concerning the criteria of the metabolic syndrome. The aim of the present work is to study the metabolic syndrome and its clinical characteristics in Marrakesh, Morocco.

## Methods

The present study consists in an epidemiological study carried out on a convenience sample of 300 patients, who visited Ibn Zohr Hospital in Marrakesh, from March to July 2018, for a routine medical check-up. These 300 subjects were chosen according to the following criteria: inclusion criteria for the participants: people aged 18 and over; both males and females and persons resident exclusively in Marrakech. Exclusion criteria for the participants: pregnant women; breastfeeding women; people with thyroid pathologies and nonresidents in the city of Marrakech. Prior to this work, we obtained an administrative authorization to carry out the study, signed by the regional delegate of the Ministry of Health, the director of the hospital and the head of the laboratory (*Note de service Régionale* N°735). After having explained the purpose of the study to the patients with the help of the nurses, they volunteered and signed the consent form to participate. They individually filled the socio-demographic information. I took the responsibility of registering the answers of illiterate subjects. Anthropometric measurements were taken in a room reserved for it. The confidentiality and privacy of the subjects were respected.

Concerning the blood tests, there was no intervention on our part. Indeed, we used the results obtained by the hospital laboratory and matched them against socio-demographic and anthropometric data, and this is done with the consent of subjects. The ethics are therefore allocated to the regional delegation of the Ministry of Health, which guarantees that those surveyed benefit from orienting and taking charge of them according to their pathology(s); if there is pathology, this will make our study ethically acceptable. The variables in which we were interested are: socio-demographic variables: age, sex, level of education, marital status and job. Anthropometric variables: weight, height, waist circumference and BMI. Biological and clinical variables: glycemia, triglyceridemia, HDL cholesterol and blood pressure. The weight in kilograms was measured using a SECA mechanical scale, with an accuracy of 0.5Kg. The size is measured by the measuring board. The BMI was calculated to assess the degree of obesity of each subject. Waist circumference was measured using a navel tape measure. Blood pressure was recorded in the position sitting after 15 minutes of rest, at two intervals of 5 minutes. These measurements were made using a standard mercury sphygmomanometer on the right arm and the average of the two measurements was recorded for comparison. However, the blood sample of the subjects was collected by the nurses in the hospital; for each fasting subject (12 to 14h fast), they took a vial of venous blood. The blood samples were taken from 8: 00 to 10: 30 in the morning. Then the blood parameters were measured by an adequate biochemistry automaton after 10 min of centrifugation.

The biological analyses were undertaken by laboratory technicians, under the supervision of the regional director of the Marrakech region. We relied on the results achieved by the Ibn Zohr hospital staff. The diagnosis of metabolic syndrome was selected according to the National Cholesterol Education Program Adult Treatment Panel III definition (NCEP-ATP III) and requires the combination of at least three of the following five criteria: waist circumference for female ≥ 88cm and for male ≥ 102cm, hypertriglyceridemia ≥ 1.5 g/L, HDL cholesterol < 0.50 g/L for female and < 0.40 g/L for male, blood pressure ≥ 130/85 mmHg, fasting glucose level ≥ 1.1 g/L (or under medical treatment of the above mentioned abnormalities) [3]. The biological and clinical analyses of the subjects were retrieved from the clinical measurement database at Ibn Zohr Hospital. These analyses concerning the targeted people are the subject of our study. To perform statistical analyses, we used SPSS (Statistical Package for Social Sciences) version 23.0. Quantitative variables were described using mean and standard deviation (SD) whereas categorical variables were described using cases numbers and percentages. Furthermore, we used the Chi-squared test X^2^ to evaluate the association between two categorical variables. The test is considered as significant when the p-value is lower than 0.05.

## Results

**Metabolic syndrome and socio-demographic characteristics:** a total of 79 people had metabolic syndrome which represents 26.3% of the total population. Table 1 shows a predominance of females with metabolic syndrome (34.9%) compared to men with (14.8%) especially in the 55 to 70 age group. The illiterates (33.8%) as well as the married subjects (25.6%) are the most affected by the metabolic syndrome (Table 1).

**Table 1:** socio-demographic characteristics by presence or absence of metabolic syndrome

	MS + (26.3%)		MS- (73.7%)		Test Khi-deux
	Number	Percentage %	Number	Percentage %	P
**Age group (years)**					< 0.001
[18-25]	0	0	13	100	
[25-40]	5	8.9	51	91.1	
[40-55]	27	29.3	65	70.7	
[55-70]	41	31.8	88	68.2	
>70	6	60.0	4	40.0	
**Gender**					< 0.001
Female	60	34.9	112	85.2	
Men	19	14.8	109	65.1	
**Level of Education**					< 0.001
Illiterate	68	33.8	133	66.2	
Primary	5	10.9	41	89.1	
Secondary	3	23.1	10	76.9	
Superior	3	7.5	37	92.5	
**Marital Status**					0.005
Single	4	8.7	42	91.3	
Divorced	9	40.9	13	59.1	
Married	43	25.6	125	74.4	
widower	23	35.9	41	64.1	
**Job**					
					0.8
With	33	26.8	90	73.2	
Without	46	26.0	131	74.0	

MS +: with Metabolic Syndrome; MS -: without metabolic Syndrome; p: statistical significance

**Metabolic syndrome and anthropometric parameters:** in our study there is a significant difference between high waist circumference, BMI and the presence of metabolic syndrome, (P = 0.001) and (P < 0.001) respectively (Table 2).

**Table 2:** anthropometric parameters by presence or absence of metabolic syndrome

	MS+ (26.3%)		MS- (73.7%)		Test Khi 2
	Number	Percentage %	Number	Percentage %	P
**Waist Measurement**					0.001
Normal	2	3.2	60	96.8	
High	77	32.4	161	67,6	
**BMI**					< 0.001
Malnutrition	0	0	2	100	
Thinness	1	12.5	7	87.5	
Normal	11	14.7	64	85.3	
Overweight	21	17.2	101	82.8	
Obesity	46	49.5	47	50.5	


BMI: Body Mass Index; MS +: with metabolic syndrome; MS -: without metabolic syndrome; p: statistical significance

**Figure 1:**
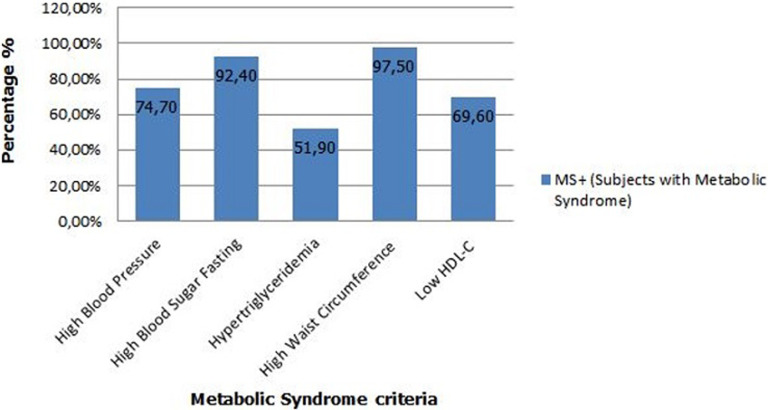
percentage of metabolic syndrome criteria

**Percentage of metabolic syndrome criteria:** the predominant parameter was the high waist circumference found in 97.5% of subjects with the metabolic syndrome, followed by hyperglycemia in 92.4%. Hypertension was found in 74.7% of cases, a low HDL-C in 69.6%; and finally hypertriglyceridemia in 51.9% of cases (Figure 1).

**Figure 2:**
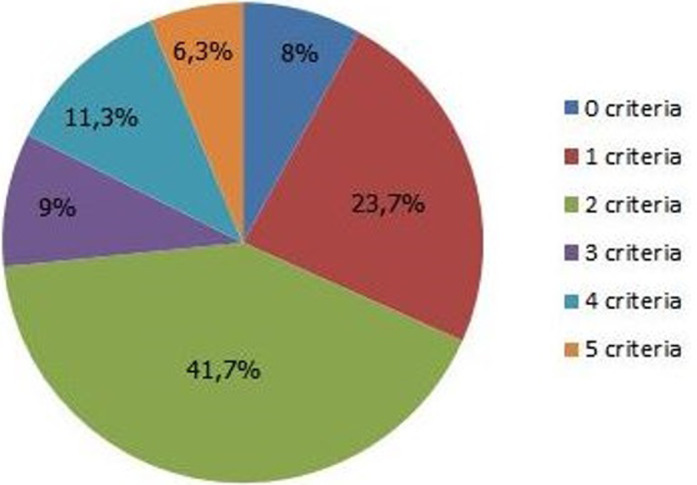
percentage of the number of metabolic syndrome criteria in the population

**Percentage of the number of metabolic syndrome criteria:** according to the NCEP-ATP III definition, 8% of our patients had no metabolic syndrome criteria, while 41.7% had two metabolic syndrome criteria. Maximum of criteria were present in 6.3% of the population (Figure 2).

**Figure 3:**
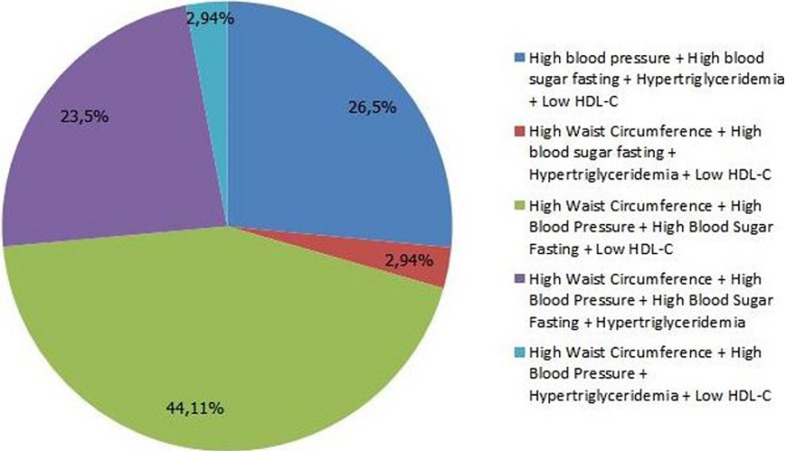
frequency of each combination of metabolic syndrome criteria

**Frequency of each combination of metabolic syndrome criteria:** in our population it was the presence of High Waist Circumference (HWC) + High Blood Pressure (HBP) + High Blood Sugar Fasting (HBSF) + Low HDL-Cholesterol (↙HDL-C) both (44.11%) the combination of metabolic syndrome´s criteria the most dominant (Figure 3).

## Discussion

**Analysis and discussion of metabolic syndrome with socio-demographic and anthropometric parameters:** according to the NCEP-ATP III definition the frequency of the metabolic syndrome in our population was 26.3% (all sexes combined): 34.9% of women and 14.8% of men. This difference due to gender was statistically significant (P < 0.001). In other studies the frequency of metabolic syndrome was reported: 8.6% Sale [4], 21.7% Casablanca [5], 17.4% Tlemcen - Algeria [6]. The percentage of metabolic syndrome in Marrakesh remains by far the highest percentage in Morocco, indeed a study was conducted in Oujda where it was 35% [7]; nevertheless, the difference between regions can be explained either by life style or by using other definition of metabolic syndrome than ours. The mean age of this study subjects was 51,6 years with a standard deviation of 13,4 years, which is approximate to a study on the epidemiological aspects of metabolic syndrome performed in Benin (51,4 ± 12,78 years old) [8].

In our population we found that the metabolic syndrome increased with age (P = 0.01). It was higher in the 55 to 70 age group: 9.7%. Indeed, the frequency of occurrence of the metabolic syndrome is high in the older ages of life, this is probably due to the fact that the elderly are sedentary and are more exposed to complications related to type 2 diabetes and obesity. This hypothesis is comforted by the DESIR study [9] which reveals an increase of the metabolic syndrome with age. The metabolic syndrome is predominantly expressed in women (24.4%) than in men (7.8%). Female dominance was observed in Casablanca (25.9% vs. 17.3%) [5], Tunis - Tunisia (37.3% vs. 23.9%) [10] and Trabzon - Turkey (31.3% vs. 21.7%) [11]. Usually, the metabolic syndrome is higher in men (25%) than women (19%). But the results of the latest ObEpi-Roche survey on overweight and obesity in France [12] show that waist circumference has been increasing for several years, with a faster progression for women than men. The incidence of the metabolic syndrome increases in the woman after the age of fifty; at menopause women are undergoing a modification of the distribution of the fats with especially a deposit around the size according to its hormonal status[13] which can explain this domination of women over men in metabolic syndrome [14].

In our study, marital status was positively associated with the metabolic syndrome (P < 0.001); the same result was observed in Erem C *et al*. [11]. The occurrence of metabolic syndrome is affected by the educational level (P = 0.005); the same result was reported in the Allal-Elasmi *et al*. [10] study. However, other studies[11, 15] indicated the opposite. Work in our study and also found in Cameroon [15], did not affect the frequency of the metabolic syndrome (P = 0.8). In our study there is a significant difference between HWC and the presence of metabolic syndrome (P = 0.001), these results are consistent with those of the literature[6]. It is actually the waist circumference that predicts better the development of a metabolic syndrome. Obesity would precede the development of the other components of the metabolic syndrome, and by aiming to reduce obesity, particularly waist circumference, we could therefore reduce the frequency of metabolic syndrome [16]. Regarding the BMI we found a positive correlation between the different levels of BMI and the presence of the metabolic syndrome (P < 0.001). Our results concord those of the American study [17] where the frequency of metabolic syndrome increased from those with normal BMI to those whom are obese.

**Analysis and discussion of metabolic syndrome´s criteria:** our study showed that the HWC 97.5% was the predominant criteria among subjects with metabolic syndrome; this is because our subjects were overweight or obese, and waist circumference increase with weight gain. Our results are consistent with other study of the metabolic syndrome and cardiovascular risk factors in Palestinian Jerusalem subjects over 20 years old [18]. In subjects with metabolic syndrome, analysis of the frequency of criteria defining this syndrome showed frequencies of 9.0%, 11.3% and 6.3% respectively for a number of criteria equal to 3, 4 and 5. 41.7% of subjects had 2 criteria, alarming result, because if dietary and lifestyle measures are not taken urgently, these subjects may develop the metabolic syndrome. A comparative study between the NHANES III data and the NHANES 1999-2000 program in the United States showed an increase of the percentage of metabolic syndrome from 24.1% to 27% this increasing is mainly related to the elevation of some parameters, mainly HWC, HBP and HBSF[16]. We have chosen 5 combinations that contain the best-represented metabolic syndrome criteria. In our population it was the presence of both HWC + HBP + HBSF + Low HDL-C (44.11%). In fact, few studies have compared the relative risk of mortality or cardiovascular events and combinations of metabolic syndrome criteria. Within the Framingham, Wilson and col cohort, no difference in risk was shown by the way the metabolic syndrome manifests itself. In contrast Guize *et al*. have identified 5 combinations of 3 criteria particularly at risk of death [17].

**Limitations of the study:** our findings cannot be extrapolated to the population of Marrakesh as the number of subjects is reduced and is not representative. The dosage of certain parameters such as CRP and micro albuminuria will also be of great interest. These parameters represent early indicators of cardiovascular risk factors. The lack of Moroccan biological and anthropometric profiles.

## Conclusion

Metabolic syndrome is a pathological entity that affects a large fraction of the population. This pathology exposes its individuals to serious complications such as cardiovascular risks and type 2 diabetes. These data should therefore encourage us to identify early at-risk groups in order to start treatment as soon as possible, but above all to encourage the application of hygienic and dietary rules which remains the best remedy and means of prevention of the metabolic syndrome.

### What is known about this topic

- Metabolic syndrome is a cluster of metabolic disorders and risk factors for cardiovascular diseases and insulin resistance;
- Given the heterogeneity of the syndrome, there is no specific treatment for it;
- Morocco, in the midst of a demographic, nutritional and epidemiological transition, suffers from the consequences of the deviation from the Mediterranean diet and the change in its lifestyle.

### What this study adds

- We have known the relation between metabolic syndrome and socio-demographic and anthropometric parameters;
- Our results inform us of the urgency of a healthy lifestyle strategy if we want to stop the progression of the metabolic syndrome and its criteria.

## References

[1] Delarue J, Allain G, Guillerm S (2006). Le syndrome métabolique. Nutrition Clinique et Métabolisme.

[2] Allali F (2017). Evolution des pratiques alimentaires au Maroc. International Journal of Medicine and Surgery.

[3] (2001). Executive Summary of The Third Report of The National Cholesterol Education Program (NCEP) Expert Panel on Detection, Evaluation, And Treatment of High Blood Cholesterol In Adults (Adult Treatment Panel III).

[4] Samara I, Bour A (2016). The prevalence of metabolic syndrome compared to physical activity in a population of Sale, a North West city of Morocco. The Swedish Journal of Scientific Research.

[5] Laraqui O, Laraqui S, Manar N, Loukili M, Deschamps F, Laraqui C (2017). Screening and prevalence of the main components of the metabolic syndrome among health care workers in Morocco. International Journal of Innovation and Applied Studies.

[6] Berrouiguet A, Brouri M (2019). Prévalence et caractéristiques du syndrome métabolique à Tlemcen: étude en population. Diabetes & Metabolism.

[7] Sellam El B, Bour A (2016). Prevalence of risk factors for cardiovascular diseases in women in Oujda (Morocco). Médecine des maladies Métaboliques.

[8] Yessoufou AG, Behanzin J, Issotina ZA, Djihoumeto E, Ahokpe M, Baba-Moussa LS (2015). Epidemiological aspects of metabolic syndrome in the obese population of Ouidah in southwestern Benin (West Africa). International Journal of Advanced Research.

[9] Balkau B, Vernay M, Mhamdil L, Novak M, Arondel D, Vol S (2003). The incidence and persistence of the NCEP (National Cholesterol Education Program) metabolic syndrome, The French DESIR study. Diabetes & Metabolism.

[10] Allal-Elasmi M, Haj Taieb S, Hsairi M, Zayani Y, Omar S, Sanhaji H (2010). The metabolic syndrome: prevalence, main characteristics and association with socio-economic status in adults living in Great Tunis. Diabetes & Metabolism.

[11] Erem C, Hacihasanoglu A, Deger O, Topbaş M, Hosver I, Ersoz HO (2008). Prevalence of metabolic syndrome and associated risk factors among Turkish adults. Trabzon MetS study Endocrine.

[12] (2009). ObEpi-Roche: 5^e^ édition de l´enquête nationale sur la prévalence de l´obésité et du surpoids en France, novembre 2009. Etude épidémiologique réalisée sur un échantillon représentatif de la population française adulte. Enquêtes réalisées par l´INSERM/l´Institut Roche de l´Obésité/TNS-SOFRES.

[13] Gambacciani M, Ciaponi M, Cappagli B, Benussi C, De Simone L, Genazzani AR (1999). Climacteric modifications in body weight and fat tissue distribution. Climacteric.

[14] Berdah J (2010). La femme est le syndrome métabolique. Revues Générales.

[15] Damaris E M, Messi Z (2018). Facteurs de risques sociodémographiques du syndrome métabolique dans une population de Yaoundé-Cameroun. International Journal Of Advanced Research.

[16] Eschwège E (2005). Le syndrome métabolique: quelle(s) définition(s) pour quel(s) objectif(s)?. Annales endocrinologie.

[17] Hansel B, Bastard J-P, Bruckert E (2011). Syndrome métabolique. Endocrinologie-nutrition.

[18] Abu Sham R, Darwazah AK, Kufri FH, Yassin IH, Torok NI (2009). Metabolic syndrome and cardiovascular risk factors among Palestinians of East Jerusalem. Eastern Mediterranean Health Journal.

